# A case report of the two big C’s; Cancer or catatonia?

**DOI:** 10.1192/j.eurpsy.2025.1783

**Published:** 2025-08-26

**Authors:** S. Kocijancic Azzaoui, J. Knific, A. Andlovic

**Affiliations:** 1 Psycho-oncology; 2 Neuro-oncology, Institute of Oncology, Ljubljana, Slovenia

## Abstract

**Introduction:**

Catatonia is a severe neuropsychiatric condition with distinct motor, behavioural, and affective abnormalities that can manifest suddenly and dramatically. While it is commonly associated with psychiatric disorders, catatonia can also emerge in the context of various medical conditions, including malignancies.

**Objectives:**

This report details the case of a 68-year-old female with lymphoma who developed sudden-onset catatonia, highlighting the complexities of diagnosing and managing this rare complication.

**Methods:**

The patient presented with symptoms of unresponsiveness and aphasia, acutely in the department just a few hours after the nurses reported, she had been confused during the night shift and had visual hallucinations. First the neurologist was consulted. Neurological examination revealed unresponsiveness to verbal or pain stimuli, flaccid muscle tonus, no plantar response, normal reflexes, staring gaze, blinking and resistance to open eyelids. Corneal and oculocephalic reflex including cardiorespiratory functions were normal. Due to the acute nature of the symptoms and history of iatrogenic subdural hygroma a trial with anticonvulsant was performed, with no improvement. CT scan, MRI scan and EEG were unremarkable. Results of the lumbar puncture ruled out any inflammation, infection or lymphoma infiltration.

**Results:**

Since the clinical findings did not correlate with test results, the neurologist consulted the psychiatrist. The patient was promptly treated with lorazepam 2 mg intramuscularly, which is the first-line treatment for catatonia. Within 2 hours of initiating treatment, there was a noticeable improvement in her responsiveness. Over the next 24 hours, she regained the ability to speak and follow commands. The decision was made to continue lorazepam 2 x 1 mg per day plus 2 x 1 mg if needed. After three days the consultation psychiatrist came to do a checkup and found the patient talking slowly and quietly, with some anxiety symptoms, but no apparent mood disorder or delusions or hallucinations. The patient was prescribed antidepressants and continued lorazepam treatment. The psychiatrist tried to decrease the lorazepam dose before the patient discharge, which resulted in worsening of her mental state. Lorazepam dose was again raised to the previous effective dose. She was discharged with sertraline 50 mg and lorazepam 2 x 1 mg. The patient had a checkup a month after being discharged. At the checkup she presented not only without any signs of catatonia, but in complete remission of any mood or anxiety or psychosis symptoms. At that point, the lorazepam dose was reduced, with the intention of keeping only the antidepressant therapy.

**Conclusions:**

Catatonia is a rare and serious complication for patients with lymphoma, and recognizing it constitute a challenge as demonstated by this case. However the lorazepam challenge test still remains a great diagnostic treatment.

**Disclosure of Interest:**

None Declared

